# Post-Operative Complications after Foramen Magnum Decompression with Duraplasty Using Different Graft Materials in Adults Patients with Chiari I Malformation: A Systematic Review and Meta-Analysis

**DOI:** 10.3390/jcm12103382

**Published:** 2023-05-10

**Authors:** Paolo Perrini, Daniele Lorenzini, Alberto Vercelli, Alessandra Perrone, Davide Tiziano Di Carlo

**Affiliations:** 1 Department of Neurosurgery, Azienda Ospedaliero Universitaria Pisana, 56124 Pisa, Italy; 2 Department of Translational Research on New Technologies in Medicine and Surgery, University of Pisa, 56126 Pisa, Italy

**Keywords:** Chiari malformation, dural graft, duraplasty, surgery, pseudomeningocele, CSF leak

## Abstract

Despite extensive investigations, the choice of graft material for reconstructive duraplasty after foramen magnum decompression for Chiari type I malformation (CMI) is still a topic of discussion. The authors performed a systematic review and meta-analysis of the literature examining the post-operative complications in adult patients with CMI after foramen magnum decompression and duraplasty (FMDD) using different graft materials. Our systematic review included 23 studies with a total of 1563 patients with CMI who underwent FMDD with different dural substitutes. The most common complications were pseudomeningocele (2.7%, 95% CI 1.5–3.9%, *p* < 0.01, I^2^ = 69%) and CSF leak (2%, 95% CI 1–2.9%, *p* < 0,01, I^2^ = 43%). The revision surgery rate was 3% (95% CI 1.8–4.2%, *p* < 0.01, I^2^ = 54%). A lower rate of pseudomeningocele was observed with autologous duraplasty when compared with synthetic duraplasty (0.7% [95% CI 0–1.3%] vs. 5.3% [95% CI 2.1–8.4%] *p* < 0.01). The rate of CSF leak and revision surgery was lower after autologous duraplasty than after non-autologous dural graft (1.8% [95% CI 0.5–3.1%] vs. 5.3% [95% CI 1.6–9%], *p* < 0.01 and 0.8% [95% CI 0.1–1.6%] vs. 4.9% [95% CI 2.6–7.2%] *p* < 0.01, respectively). Autologous duraplasty is associated with a lower rate of post-operative pseudomeningocele and reoperation. This information should be considered when planning duraplasty after foramen magnum decompression in patients with CMI.

## 1. Introduction

Foramen magnum decompression with duraplasty (FMDD) is the most common treatment for adult patients with isolated Chiari type I malformation (CMI) and with syringomyelia-CMI complex [1]. In fact, clinical studies reported that FMDD had higher rate of clinical improvement, lower rates of revision surgery and greater reduction in syrinx size when compared with FMD by bony decompression alone [2,3,4]. The rate of post-operative complications after surgical treatment of CMI is uncertain in the literature and ranges between 3% and 40%, consisting primarily of pseudomeningocele (PSMC), CSF leak, aseptic meningitis and wound infection [5,6,7]. The effective role of the types of dural grafts on the occurrence of post-operative complications is not yet defined. A recent meta-analysis found that autograft was associated with the lowest rate of complications when compared to collagen-based grafts, allograft and nonautologous graft materials [8]. However, the generalizability and the strength of these findings were limited by the fact the patient cohort included both pediatric and adult patients. We conducted a systematic review and meta-analysis of the literature investigating the occurrence rate of complications after FMDD in adults and analyzed the effect of graft materials on post-operative outcome.

## 2. Materials and Methods

### 2.1. Literature Search

A comprehensive literature search of PubMed, Cochrane Library and Ovid SCOPUS was conducted for studies published from January 1990 to December 2022. PRISMA guidelines (Preferred Reporting Items for Systematic Reviews and Meta-analysis [9]) were followed. The key words, detailed search strategy and the inclusion criteria are reported in Table 1. In cases of overlapping patient populations, only the series with the largest number of patients or most detailed data were included. Two independent readers (A.V. and D.L.) screened articles in their entirety to determine eligibility for inclusion. A third author solved discrepancies (D.D.C.).

### 2.2. Data Collection

From each study, we extracted the following: (1) demographic data; (2) mean follow-up; (3) surgical technique and graft material; (4) post-operative PSMC; (5) post-operative CSF leak; (6) post-operative infections; (7) number of reoperations (due to the followings: PSMC, CSF leak, post-operative infections); and (8) post-operative aseptic meningitis. Graft materials were classified as follows: autologous, synthetic and biological nonautologous (allograft and xenograft).

### 2.3. Outcomes

The primary objective of this systematic review and meta-analysis was to determine the rate of PSMC, CSF leak, reoperation, aseptic meningitis and post-operative infections among adult CMI population treated with FMDD, and to examine the impact of different graft material on the outcomes.

### 2.4. Quality Scoring

A modified version of the Newcastle–Ottawa Scale [10] was used for the quality assessment of the included studies. The quality assessment was performed by two authors independently (A.V. and D.L.). A third author solved discrepancies (D.D.C.). Studies rated with a NOS ≥ 6 were considered as “high quality.”

### 2.5. Statistical Analysis

Inter-observer agreement was tested with Cohen’s kappa coefficient (k). The Wald method was used to calculate confidence intervals (CI) for event rates. In order to assess the heterogeneity of the data, the Higgins index (I^2^) [11] was used, in which I^2^ > 50% suggests substantial heterogeneity. DerSimonian and Laird random-effects model was subsequently applied [12]. Predictors of outcome were analyzed with random meta-regression. The graphical representation of the meta-analysis was performed by forest plot. Differences were considered significant at *p* < 0.05. Statistical analyses were performed with SPSS version 23 (SPSS Inc. SPSS^®^, Chicago, IL, USA), with ProMeta version3 (Internovi, Cesena, Italy) and OpenMeta [Analyst] (http://www.cebm.brown.edu/openmeta/, accessed on 1 December 2022).

## 3. Results

### 3.1. Literature Review

**Figure 1:**
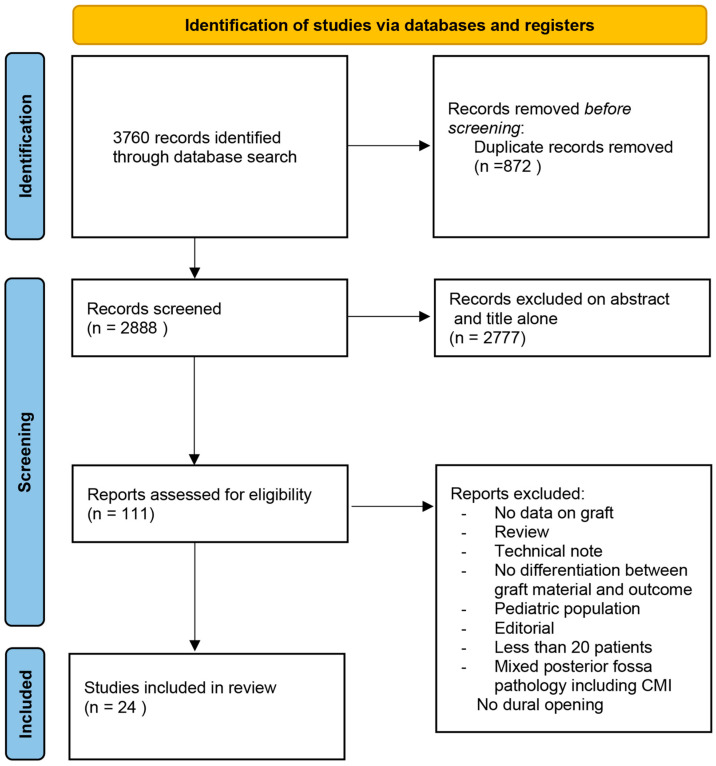
PRISMA diagram detailing the specifics of the systematic literature review.

Studies included in our systematic review are summarized in Table 2. Inter-observer agreement was 0.82. The search flow diagram is shown in Figure 1.

Twenty-three studies and 1541 adult patients with CMI that underwent FMDD were analyzed in this review.

### 3.2. Quality of Studies

All articles presented a retrospective design. All studies were rated “high quality”. Inter-observer agreement was 0.88.

### 3.3. Demographic Data and Clinical Characteristics

The mean age of the included patients ranged between 31 to 50 years old, and the proportion of female patients was 72.1% (95% CI 69.9–74.3%). Associated syringomyelia was reported in 56.9% of cases (95% CI 54.2–59.4%). FMDD was completed with autologous graft in 602 patients (38.4%, 95% CI 36.4–41.3%), whereas a biological nonautologous graft or a synthetic graft were used in 331 (21%, 95% CI 19.4–23.5%) and 617 patients (39.8%, 95% CI 37.4–42.3%), respectively (Table 3).

### 3.4. Post-Operative Complications Related to Graft Material

**Figure 2:**
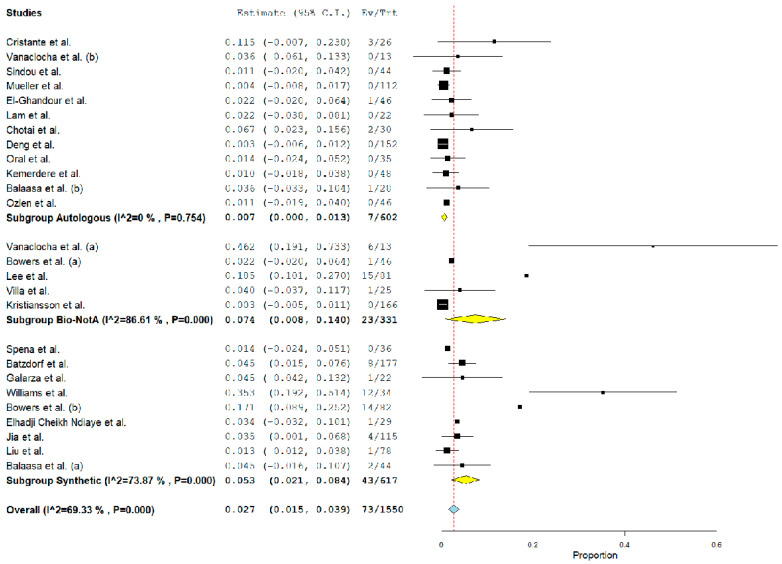
Forest plot detailing the pooled rate and 95% confidence intervals for the rate of post-operative PSMC accounted for graft material. Yellow diamond: sub-group pooled rate, blue diamond: overall pooled rate. The subgroup analysis demonstrated that the rate of PSMC was higher among biological nonautologous and synthetic groups [13,14,15,16,17,18,19,20,21,22,23,24,25,26,27,28,29,30,31,32,33,34,35].

**Figure 3:**
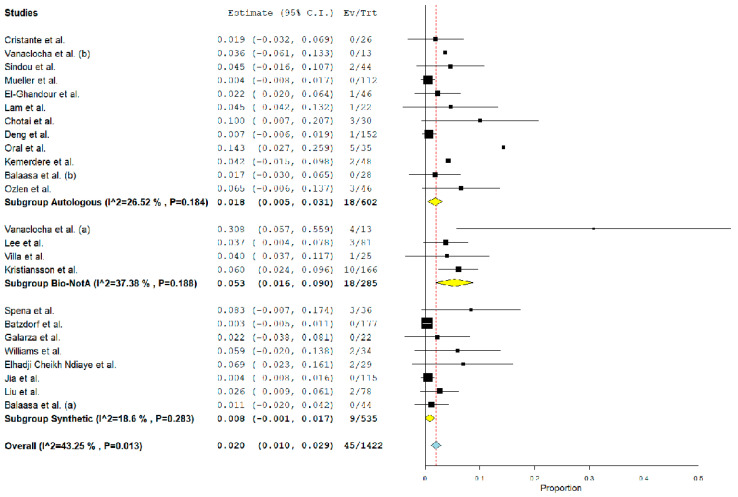
Forest plot detailing the pooled rate and 95% confidence intervals for the rate of post-operative CSF leak accounted for graft material. Yellow diamond: sub-group pooled rate, blue diamond: overall pooled rate. The subgroup analysis demonstrated that the rate of CSF leak was higher among biological nonautologous graft group [13,14,15,16,17,18,19,20,21,22,23,24,25,26,27,28,29,30,31,32,33,34,35].

**Figure 4:**
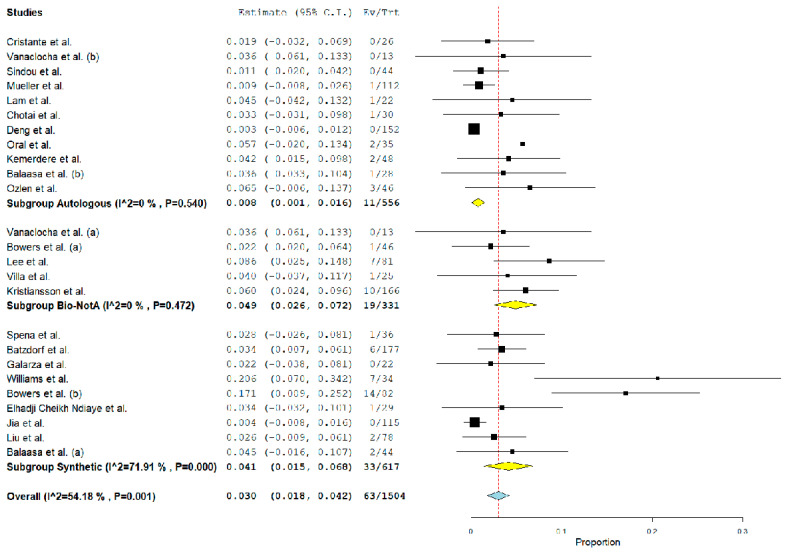
Forest plot detailing the pooled rate and 95% confidence intervals for the rate of reoperation accounted for graft material. Yellow diamond: sub-group pooled rate, blue diamond: overall pooled rate. The subgroup analysis demonstrated that the rate of PSMC was higher among biological nonautologous and synthetic groups [13,14,15,16,17,18,19,20,21,22,23,24,25,26,27,28,29,30,31,32,33,34,35].

Overall, the rate of post-operative PSMC was 2.7% (95% CI 1.5–3.9%, *p* < 0.01, I^2^ = 69%) (Figure 2). Furthermore, the rate of CSF leak and the reoperation rate were 2% (95% CI 1–2.9%, *p* < 0.01, I^2^ = 43%) (Figure 3) and 3% (95% CI 1.8–4.2%, *p* < 0.01, I^2^ = 54%) (Figure 4), respectively. Wound infection occurred in 0.6% (95% CI 0.2–1%, *p* < 0.01, I^2^ = 0%), and bacterial meningitis in 0.8% (95% CI 0.4–1.3%, *p* < 0.01, I^2^ = 0%). Moreover, aseptic meningitis was reported in 0.6% of cases (95% CI 0.2–1%, *p* < 0.01, I^2^ = 3.9%). When considering graft material, PSMC was significantly more common among the synthetic graft population when compared with autologous grafts (5.3% vs. 0.7%, *p* < 0.01). On the other hand, although the overall rate was higher among biological nonautologous grafts, the statistical analysis failed to find a significant difference between biological nonautologous and autologous grafts (0.7% vs. 7.4%, *p* = 0.9). Furthermore, post-operative CSF leak and the rate of reoperation were significantly more common among biological nonautologous grafts than in autologous FMDD (5.3 vs. 1.8%, < 0.01, and 4.9% vs. 0.8%, < 0.01, respectively). No differences were observed between synthetic and autologous grafts for the same outcomes (0.8% vs. 1.8%, and 4.1% vs. 0.8%) (Table 4). Similarly, our analysis demonstrated no significant association between the rate of post-operative aseptic meningitis, bacterial meningitis, wound infection and the graft material (detailed data in Table 4).

### 3.5. Study Heterogeneity and Sensitivity Analysis

**Figure 5:**
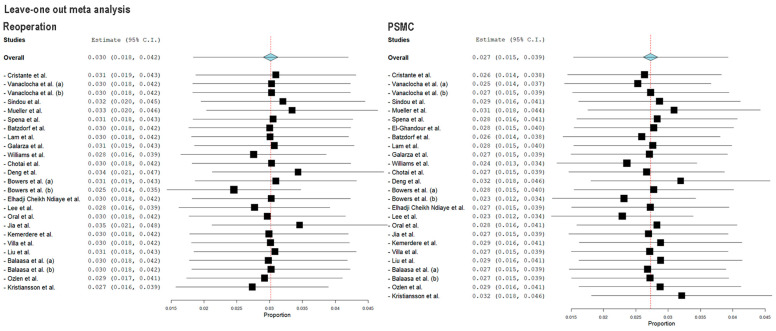
Leave-one-out sensitivity analysis for reoperation rate and post-operative PSMC ensuing FMDD. Blue diamond: overall pooled rate. The analysis shows that no individual study significantly affects the outcome [13,14,15,16,17,18,19,20,21,22,23,24,25,26,27,28,29,30,31,32,33,34,35].

I^2^ was greater than 50% for the rate of reoperation and post-operative PSMC (I^2^ = 54 and 69%, respectively). Subgroup analysis showed that the biological nonautologous group and the synthetic group carried a high heterogeneity (86.6 and 73.9%, respectively). Similarly, for reoperation rate, the synthetic group results increased the overall I^2^. In both cases, small studies increased the heterogeneity on the overall analysis (Figure 2 and Figure 4). Nonetheless, leave-one out meta-analysis showed that no single study significantly affected the outcome *p* < 0.01 (Figure 5).

## 4. Discussion

The occurrence of post-operative CSF-related complications, including PSMC, CSF leak and meningitis is one of the main concerns after FMDD for CMI. Although several substitute materials for dura have been proposed in recent years, including autograft, synthetic graft, xenograft and allograft, the choice of the dural graft after FMD is still based on the experience of the surgeon and is not supported by scientific evidence. Pooling the results of 23 studies, our analysis provides representative data on the post-operative complications after FMDD in adults using different graft materials and can give some evidence supporting the selection of dural graft for duraplasty in adults. Our meta-analysis of 1541 adult patients treated with FMDD found a low rate of CSF complications after FMDD. The overall rate of PSMC was 2.7%, and the rate of CSF leak and reoperation were 2% and 3%, respectively; the rate of wound infection, bacterial meningitis and aseptic meningitis were below 1% each. Although the current literature is heterogeneous on this topic, our results agree with previous meta-analyses that reported similar rate of post-operative complications [2,8].

One of the most relevant findings of our study is that autograft was associated with a significantly lower rate of PSMC when compared to synthetic grafts. In addition, autograft was associated with lower PSMC rate when compared to other biological grafts, but this finding did not reach statistical significance. The occurrence of PSMC is generally underreported in the literature. In a series of 371 FMDD, Klekamp reported post-operative complications in roughly 22% of operations, but no PSMC was encountered [7]. In our analysis the rate of PSMC ranged from 1% to 36%. This wide range is probably related to the way this complication is diagnosed. Recently, Balasa et al. [33] used MRI to detect the presence of even barely visible PSMC in 35.5% of patients, of whom only 11% required revision surgery. In fact, the occurrence of PSMC can remain asymptomatic or can cause symptoms inducing pain, hydrocephalus or persistent syringomyelia in long-term follow-up [36,37].

In our study, CSF leak and revision surgery were significantly lower after autologous graft when compared to other biological dural substitutes. There was no significant difference in the rates of CSF leak and revision surgery for autograft versus synthetic grafts. CSF leak is the most common complication after FMDD and is related to different factors, including the type of dural graft, the ability to perform a watertight dural closure, the tight closure of the muscular layer and the use of tissue sealants [7,38]. In fact, it has been reported that tissue sealants used in combination with a dural graft may increase the risk of CSF leak [5]. Data of suture types, closure technique and types of sealants were sometimes not available in the included studies and were not investigated in our meta-analysis. Our analysis confirms the efficacy of autologous dural graft for duraplasty after FMD. Autologous graft is nonimmunogenic and has enough strength and stretch to create a watertight closure, thereby reducing post-operative CSF-related complications. These aspects, combined with the fact that it is inexpensive, make it a graft with many ideal characteristics for dural closure. In agreement with these data, in the recent International Consensus Document on diagnosis and treatment of Chiari malformation and syringomyelia [1], the artificial graft was rejected by most of the panelists in favor of autologous graft and allografts. Although our study found significant reduction of CSF leak and revision surgery when autograft is compared with nonautologous graft, this topic is still matter of discussion in the literature. A potential drawback of autologous dural graft is post-operative pain. However, a recent multicentric study found that autograft and nonautologous graft presented no differences for post-operative pain [38].

In our study, no significant association was found between the graft and the occurrence of aseptic meningitis, bacterial meningitis and wound infection. Aseptic meningitis after FMDD is believed to result from a local inflammatory reaction due to the dural graft used and/or the sealant. However, the source of inflammation and role of the graft material remain under investigation.

In this study we provided the largest overview of data regarding complications for different types of dural grafts used for duraplasty after FMD in adult patients. Large prospective, randomized studies comparing various dural graft materials are required to characterize outcomes for different graft types. Although retrospective data are low in quality, our meta-analysis provides some evidence to guide the operating surgeon in selecting the type of dural graft for duraplasty after FMD. However, since the differences in complication rate are small, our results should be taken cautiously.

### Limitation of the Study

Our meta-analysis has limitations. Series are often retrospective studies and small, single institution experiences. Due to the small number of cases, the comparison between subgroups may not provide a comprehensive representation of outcomes differences among different dural grafts. Some important surgical information (e.g., the types of sealants) and data on how post-operative complications were assessed (e.g., recognition of PSMC on MRI) were missing in some studies and were not included in our analysis. Similarly, no sufficient data on steroid use in the perioperative time frame were available.

## 5. Conclusions

Our meta-analysis found that autologous duraplasty is associated with a lower rate of post-operative pseudomeningocele and reoperation when compared to synthetic and other biological grafts. In addition, autograft had lower CSF leak than other biological grafts. This information should be considered when planning duraplasty after foramen magnum decompression in patients with CMI.

## Figures and Tables

**Table 1:** Search syntax.

PubMed Search Accessed on December 2022 (1801 Articles)	SCOPUS Search Accessed on December 2022 (1942 Articles)	Cochrane Library Search Accessed on December 2022 (17 Articles)
(((chiari[Title/Abstract]) AND (I[Title/Abstract])) AND (malformation[Title/Abstract]) AND (2000/1/1:2022/11/30[pdat])) AND ((“1992/01/01”[Date–Publication]: “3000”[Date–Publication]))	(TITLE-ABS-KEY (chiari) AND TITLE-ABS-KEY (i) AND TITLE-ABS-KEY (malformation)) AND PUBYEAR > 1991 AND (LIMIT-TO (PUBSTAGE, “final”)) AND (LIMIT-TO (DOCTYPE, “ar”))	(chiari):ti,ab,kw AND (I):ti,ab,kw AND (malformation):ti,ab,kw” (Word variations have been searched)
Inclusion criteria	Exclusion criteria	
(1) series detailing surgical treatment and post-operative complications after FMDD for CMI; (2) series with dural graft distinction; (3) series with more than 20 patients.	(1) case reports; (2) review articles; (3) conference abstract; (4) technical notes; (5) studies published in languages other than English with no available English translations; (6) studies with overlapping patient population; (7) pediatric populations; (8) series with outcome not accounted for graft material	

**Table 2:** Summary of the studies included in the systematic review.

Study	Year	N° Patients	Syrinx (%)	Age (Mean)	Age (Range)	M	F	FU Mean ^a^	FU Range ^a^
Cristante et al. [13]	1994	26	21 (80)	50.4	35–65	13	13		5–74
Vanaclocha et al. [14]	1997	26			19–38	12	14	27	6–58
Sindou et al. [15]	2002	44	29 (66)	40		13	31	50	13–120
Mueller et al. [16]	2005	112	22 (25)	40	17–70	8	104		
Spena et al. [17]	2010	36	36 (100)	40.4	18–68	17	19	40	16–72
El-Ghandour et al. [18]	2011	46	32 (70)	37.4	19–56	21	25	88	
Batzdorf et al. [19]	2013	177	97 (55)	37.9	15–78	45	132	22.125	2–155
Lam et al. [20]	2013	22		37.3	21–61	4	18	3	3
Galarza et al. [21]	2013	22	7 (32)	38.95	18–65	10	12	18	12–32
Williams et al. [22]	2013	34		38.7		6	28	3	3
Chotai et al. [23]	2014	30	7 (23)	37	20–67	2	28	27.5	5–72
Deng et al. [24]	2015	152	112 (74)	39.2	18–60	52	100	72	
Bowers et al. [25]	2015	119	13 (11)	33.98		23	96	22.7	
Elhadji Cheikh Ndiaye et al. [26]	2017	29		39	19–66	12	17	20	3–96
Lee et al. [27]	2017	81		38.56	18–74	10	71	10.7	
Oral et al. [28]	2018	35	17 (49)		22–56	21	14	12	12
Jia et al. [29]	2019	115	115 (100)	43.4		35	80	6	6
Kemerdere et al. [30]	2019	48	21 (44)	38.8	18–69	14	33.984	30.24	6–108
Villa et al. [31]	2019	25	12 (28)	39.2	19–66	10	15	33	3–60
Liu et al. [32]	2020	78	78 (100)	40.6		36	42	20.3	18–60
Balasa et al. [33]	2021	72	54 (75)	41.9	18–66	16	54	67.3	3–187
Ozlen et al. [34]	2021	46	24 (52)	37.5		11	35	83.5	
Kristiansson et al. [35]	2022	166	70 (42)	31	21–44	37	129	4.9	4–6.6

FU follow-up. ^a^ months.

**Table 3:** Demographic data and graft characteristics.

	Raw Data	Rate (95% CI)	N° of Articles
**Demographic data**			
N patients included in the analysis	1541		23
Female patients	1111/1541	72.1% (69.6–74.3%)	23
Mean age (range)	31–50.4	–	21
Associated syringomyelia	767/1349	56.9% (54.2–59.4%)	18
**Graft Material**			
Autologous	602/1550 ^b^	38.4% (36.4–41.3%)	12
Biological nonautologous ^a^	331/1550 ^b^	21.4% (19.4–23.5%)	5
Synthetic	617/1550 ^b^	39.8% (37.4–42.3%)	9

^a^ Kristiansson et al. [35] reported the use of sutured biological patch with addition of an overlayed synthetic patch; ^b^ Bowers et al. [25] reported 128 procedures on 119 patients.

**Table 4:** Summary of post-operative complications accounted for graft material.

	Raw Data	Rate (95% CI), *p*-Value	Heterogeneity	Meta-Regression (*p*-Value)
**Pseudomeningocele**				
Autologous	7/602	0.7% (0–1.3%). 0.04	0%	–
Biological ^a^	23/331	7.4% (0.8–14%). 0.03	86.6%	0.9
Synthetic	43/617	5.3% (2.1–8.4%). <0.01	73.9%	**<0.01**
**CSF leak**				
Autologous	18/602	1.8% (0.5–3.1%). <0.01	26%	–
Biological ^a^	18/285	5.3% (1.6–9%). <0.01	37%	**<0.01**
Synthetic	9/535	0.8% (0–1.7%). 0.08	18%	0.3
**Reoperation**				
Autologous	12/556	0.8% (0.1–1.6%). 0.03	0%	–
Biological ^a^	19/331	4.9% (2.6–7.2%). <0.01	0%	**<0.01**
Synthetic	33/617	4.1% (1.5–6.8%). <0.01	71.9%	0.3
**Aseptic meningitis**				
Autologous	3/624	0.8% (0–1.6%). 0.04	0%	–
Biological ^a^	12/285	4.9% (0–10.8%). 0.1	77%	0.8
Synthetic	6/535	0.5% (0–1.2%). <0.01	1%	0.8
**Bacterial Meningitis**				
Autologous	10/602	1.2% (0.3–2%). <0.01	0%	–
Biological ^a^	6/285	2.1% (0.4–3.7%). 0.01	0%	0.3
Synthetic	5/535	0.05% (0.1.1%) <0.01	0%	0.2
**Wound Infections**				
Autologous	10/602	0.9% (10–1.6%). <0.01	0%	–
Biological ^a^	1/285	0.4% (0–1.1%). 0.01	0%	0.4
Synthetic	6/535	0.6% (0–1%). <0.01	0%	0.6

^a^ Biological nonautologous.

## Data Availability

Data sharing not applicable.
